# Expression of Castor LPAT2 Enhances Ricinoleic Acid Content at the *sn*-2 Position of Triacylglycerols in Lesquerella Seed

**DOI:** 10.3390/ijms17040507

**Published:** 2016-04-06

**Authors:** Grace Q. Chen, Harrie van Erp, Jose Martin-Moreno, Kumiko Johnson, Eva Morales, John Browse, Peter J. Eastmond, Jiann-Tsyh Lin

**Affiliations:** 1 Western Regional Research Center, Agricultural Research Service, U.S. Department of Agriculture, 800 Buchanan St., Albany, CA 94710, USA; kumiko.johnson@ars.usda.gov (K.J.); evadesalta@gmail.com (E.M.); jiann.lin@ars.usda.gov (J.-T.L.); 2 Institute of Biological Chemistry, Washington State University, Pullman, Washington, DC 99164, USA; harrie.van-erp@rothamsted.ac.uk (H.E.); jab@wsu.edu (J.B.); 3 Department of Plant Biology and Crop Science, Rothamsted Research, Harpenden, Hertfordshire AL5 2JQ, UK; jmartinm83@gmail.com (J.M.-M.); peter.eastmond@rothamsted.ac.uk (P.J.E.)

**Keywords:** lesquerella, *Physaria fendleri*, lysophosphatidic acid acyltransferase, hydroxy fatty acid, seed triacylglycerol, metabolic engineering

## Abstract

Lesquerella is a potential industrial oilseed crop that makes hydroxy fatty acid (HFA). Unlike castor its seeds are not poisonous but accumulate lesquerolic acid mostly at the *sn*-1 and *sn*-3 positions of triacylglycerol (TAG), whereas castor contains ricinoleic acid (18:1OH) at all three positions. To investigate whether lesquerella can be engineered to accumulate HFAs in the *sn*-2 position, multiple transgenic lines were made that express castor lysophosphatidic acid acyltransferase 2 (*RcLPAT2*) in the seed. RcLPAT2 increased 18:1OH at the *sn*-2 position of TAGs from 2% to 14%–17%, which resulted in an increase of tri-HFA-TAGs from 5% to 13%–14%. Our result is the first example of using a LPAT to increase ricinoleic acid at the *sn*-2 position of seed TAG. This work provides insights to the mechanism of HFA-containing TAG assembly in lesquerella and directs future research to optimize this plant for HFA production.

## 1. Introduction

Lesquerella (*Physaria fendleri*, formerly *Lesquerella fendleri* (Gray) Wats.) (Brassicaceae) [1] is valued for the unusual hydroxy fatty acids (HFAs) in its seed oil. The major HFA in lesquerella is lesquerolic acid (20:1OH) [2], comprising 55%–60% of total seed fatty acids (FAs) [3,4,5,6,7,8]. The main source of HFAs is castor (*Ricinus communis*), 90% of its seed oil is ricinoleic acid (18:1OH). 18:1OH is used as a feedstock for the manufacture of a range of products, such as lubricants, plasticizers and surfactants [9]. However, castor cultivation is limited by the presence of the toxin ricin in its seed. Lesquerella seeds do not contain ricin and therefore its oil provides a safe source of HFAs. Efforts have been made to improve lesquerella through plant breeding [4]. Since lesquerella can be transformed by *Agrobacterium* [10], genetic engineering also provides an alternative means to improve this potential crop.

**Figure 1:**
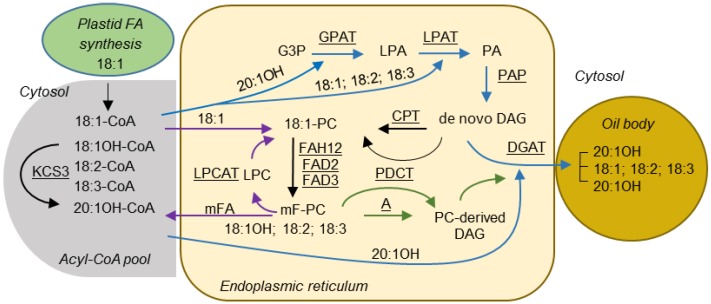
Proposed pathways for TAG biosynthesis in lesquerella seed. Blue arrows indicate reactions involved in Kennedy pathway. Purple arrows indicate reactions involved in acyl editing. Green arrows indicate reactions involved in PC-derived DAG synthesis. The rest of reactions are indicated by black arrows. Enzymes catalyzing these reactions are underlined. Fatty acid numerical symbols are described as in Table 2. Abbreviations: A, DAG production by the reverse reaction of CPT, phospholipase C or phospholipase D plus PAP; G3P, glycerol-3-phosphate; LPA, lysophosphatidic acid; PA, phosphatidic acid; LPC, lysophosphatidylcholine; PC, phosphatidylcholine; DAG, diacylglycerol; TAG, triacylglycerol; GPAT, glycerol 3-phosphate acyltransferase; LPAT, lysophosphatidic acid acyltransferase; PAP, phosphatidic acid phosphatase; FAH12, Δ12 oleic acid hydroxylase; FAD2, Δ12 oleic acid desaturase; FAD3, Δ15 (ω-3) linoleic acid desaturase; KCS3, 3-ketoacyl-CoA synthase 3; LPCAT, lysophosphatidylcholine acyltransferase; CPT, CDP-choline:DAG cholinephosphotransferase; PDCT, PC:DAG cholinephosphotransferase; DGAT, diacylglycerol acyltransferase.

Seed oils or triacylglycerols (TAGs) are storage lipids in which FAs are esterified to each of the three hydroxy groups of a glycerol backbone. FAs are synthesized in the plastid, exported and converted to acyl-coenzyme As (acyl-CoAs) for the synthesis of TAG in the endoplasmic reticulum (ER) [11,12]. The synthesis of TAG requires the Kennedy pathway [12,13,14,15] (Figure 1). The first acylation reaction is catalyzed by a glycerol-3-phosphate acyltransferase (GPAT) and yields lysophosphatidic acid (LPA), which in turn is acylated by a lysophosphatidic acid acyltransferase (LPAT) to produce phosphatidic acid (PA). PA is then converted to 1,2-*sn*-diacylglycerol (DAG) by PA phosphatase (PAP). Finally, a third FA is esterified to the *sn*-3 position of DAG by 1,2-*sn*-diacylglycerol acyltransferase (DGAT) to produce TAG. Alternatively, FAs can be directly incorporated into phosphatidylcholine (PC) by the reactions of the acyl editing cycle [16,17] (Figure 1). These reactions are catalyzed by the forward and reverse reactions of lyso-PC acyltransferase (LPCAT) to yield acyl-CoA, or by a phospholipase A-type activity to yield a free FA that then is activated to acyl-CoA. Because PC is the substrate for FA-modifying enzymes such as desaturases and hydroxylases, rapid de-acylation and re-acylation of PC causes the acyl-CoA pool to be enriched with modified FAs (mFA), which can subsequently be used for TAG synthesis. FAs can also be acylated to the *sn*-3 position of DAG by Phospholipid: DAG acyltransferase (PDAT) [18]. In addition, metabolic labeling experiments showed that there are two pools of DAG, *de novo* DAG and PC-derived DAG [19,20] (Figure 1). The *de novo* synthesis of DAG involves the Kennedy pathway, as described above. In the PC-derived DAG pathway, *de novo* DAG is used to synthesize PC. Removal of the head group of PC by PC:DAG cholinephosphotransferase (PDCT) can generate PC-derived DAG [21,22] (Figure 1). Alternatively, PC-derived DAG can be produced by the reverse action of CDP-choline: DAG cholinephosphotransferase (CPT) [23], or a lipase-based mechanism using phospholipase C, or phospholipase D plus PAP. As FAs in PC can be modified, the conversion of PC into DAG also provides a means to increase the amount of mFAs in TAG.

Castor TAG contains 90% 18:1OH, over 70% of the TAGs having all three *sn* positions esterified with 18:1OH [24]. The 18:1OH is synthesized by hydroxylation of 18:1, which is esterified to the *sn*-2 position of PC, efficiently released and activated to 18:1OH-CoA, and then redistributed into the *sn*-1, *sn*-2, and *sn*-3 positions of TAG [25,26]. Biochemical evidence indicates that in lesquerella, 18:1OH is initially synthesized by hydroxylation of 18:1 at the *sn*-2 position of PC, released and activated to 18:1OH-CoA. Subsequently, 18:1OH-CoA is rapidly elongated to 20:1OH-CoA for TAG synthesis [27,28]. The lesquerella gene *PfKCS3* responsible for the elongation of 18:1OH-CoA to 20:1OH-CoA has been identified [29] (Figure 1). In addition to 18:1OH and 20:1OH, lesquerella seed accumulates a low level of densipolic acid (18:2OH) and auricolic acid (20:2OH), which were suggested to be synthesized by a microsomal ∆-15 desaturase [27,28].

Arabidopsis, which does not accumulate HFAs, has been used as a model to study genes associated with HFA synthesis [30]. A key gene encoding the oleate 12-hydroxylase (FAH12) was first isolated from castor (*CFAH12* or *RcFAH12*) [31], and then from lesquerella (*PfFAH12*) [32]. Expression of *RcFAH12* or *PfFAH12* in Arabidopsis led to accumulation of HFAs to no more than 17% in seed oil [32,33,34,35,36]. To boost HFA production, additional genes from castor have been co-expressed with *RcFAH12* in Arabidopsis, including *RcDGAT2* [37], *RcPDAT1A* [38], or *RcPDAT1-2* [39], and *RcPDCT* [21]. The overexpression of RcDGAT2 and RcPDAT1A resulted in an increase in seed HFA content from 17% to 23%–28% [38]. Co-expression of castor DGAT, PDAT and PDCT, while simultaneously suppressing the expression of endogenous acyltransferases, may increase the flux of HFA into TAG through more efficient utilization of HFA-containing substrates, such as HFA-CoA, HFA-DAG, and HFA-PC, in their pathways [20,21,38,39,40].

Lesquerella TAGs contain ~60% 20:1OH and almost all of it is esterified to the *sn*-1 and *sn*-3 positions [3,7] (Figure 1). The absence of HFAs at the *sn*-2 position of TAG might be caused by the selectivity of LPATs in lesquerella for common FAs. This is supported by the fact that LPAT enzymes from plants are some of the most stringent acyltransferases regarding substrate discrimination [41]. Based on several studies of plant microsomal (putative ER-localized) LPATs, Frentzen (1998) proposed the presence of at least two distinct classes of LPATs, class-A and -B, sharing little sequence homology between them. The class-A LPATs had selectivity for unsaturated C_18_ acyl groups, and they are ubiquitously expressed in higher plants for membrane lipid and TAG synthesis [41]. The class-B LPATs are present in *Limnanthes* [42] and *Cocos* [43]. Their seed oils are enriched with the unusual FAs (UFAs), erucic acid (22:1) and lauric acid (12:0), respectively, at the *sn*-2 position of TAGs. Their corresponding members of class-B LPATs possessed substrate preference for UFAs and were used to improve FA composition in industrial oilseeds by genetic engineering [41,44,45,46]. Interestingly, a yeast LPAT sharing little homology to plant LPATs also has preference for esterifying unusual very long chain FAs at the *sn*-2 position of seed TAGs [47]. Most plant species do not have a representative of the class-B gene, including Arabidopsis where five *LPATs* have been identified [48,49]. Arabidopsis *LPAT2* (*AtLPAT2*) is the lone gene that encodes the ubiquitous and ER-located LPAT, and is thus suggested to be the key enzyme involved in membrane and seed oil biosynthesis [49]. Recently, a highly seed-expressed *LPAT* was identified from *Sterculia foetida* whose seeds accumulate up to 60% of desaturated cyclopropane FA [50]. *SfLPAT* showed strong homology to *AtLPAT2* and enabled the incorporation of the unusual cyclopropane FA at the *sn*-2 position of LPA in transgenic Arabidopsis [50]. In castor, whole genome data [51] allowed the identification and analysis of the LPAT multigene family [51,52,53,54,55]. Among them, two genes, castor lysophosphatidic acid acyltransferase 2 (*RcLPAT2*) and castor lysophosphatidic acid acyltransferase B (*RcLPATB*), representing class-A and -B respectively, were found to express highly in seed tissues and thus were characterized in more detail [55]. Spatial and temporal gene expression profiling indicated that *RcLPAT2* exhibited a generalized constitutive pattern typical to that of class-A members. *In vitro* enzyme assays indicated that RcLPAT2 had a preference for 18:1OH-CoA when *sn*-1-ricinoleate-*sn*-glycerol-3-phosphate (18:1OH-LPA) was used as an acyl acceptor, whereas when *sn*-1-oleoyl-*sn*-glycerol-3-phosphate (18:1-LPA) was used as the acyl-acceptor, RcLPAT2 preferred 18:1-CoA as a substrate [55]. These results suggest that RcLPAT2 might participate in both TAG and membrane glycerolipid synthesis. However, unlike the *LPATBs* from *Limnanthes* and *Cocos*, the expression of *RcLPATB* was not restricted to seeds as it was also expressed at a significant level in leaf, stem and floral organs. However, *in vitro* assays indicated that RcLPATB had a low enzyme activity and a broad specificity for acyl-CoAs compared with that of RcLPAT2. RcLPATB catalyzed the incorporation of saturated FAs, 12:0–16:0, at rates surpassing those of monounsaturated FAs including 18:1-CoA and 18:1OH-CoA, independently of the acceptors 18:1-LPA or 18:1OH-LPA [55]. Therefore, it remains questionable if RcLPATB is involved in HFA-containing TAG synthesis. We note that before the completion of castor genome sequencing, expression of a castor gene (GenBank ID, EU391594) designated as a “putative castor *LPAT*” [37] did not change HFA composition in transgenic Arabidopsis expressing *RcFAH12*. The sequence (EU391594) shows no significant similarity to RcLPAT2 (GenBank ID, JQ796917) and is now annotated as a putative castor 1-acyl-*sn*-glycerol-3-phosphate acyltransferase zeta precursor [51] (GenBank ID, XP_002525812).

Castor oil has long been used by industry as a raw material for manufacturing numerous established products. If lesquerella oil can be reengineered to resemble castor oil, this would provide an alternative source of castor oil that is safe, cost-competitive, and readily adaptable by existing industrial technologies. Increasing the levels of tri-HFA-TAG and 18:1OH, are key steps towards achieving this goal. We therefore over-expressed RcLPAT2 in lesquerella seeds. Analysis of seed TAGs suggests that RcLPAT2 is able to acylate the *sn-*2 position of LPA with 18:1OH and as a result the amounts of 18:1OH and tri-HFA-TAGs were increased. Additional genetic modifications for engineering a castor-oil producing lesquerella crop are discussed. To our knowledge this is the first time that castor *RcLPAT2* has been expressed in another oilseed to modify HFA content and also the first example of metabolic engineering in lesquerella.

## 2. Results

### 2.1. Castor Lysophosphatidic Acid Acyltransferase 2 (RcLPAT2) Increased 18:1OH Accumulation in Transgenic Seeds

We have produced selfed T_1_ seeds from 16 independent primary transgenic plants (T_0_); expressing *RcLPAT2* under the control of the seed-specific napin (napA) promoter. Analysis of transgene *RcLPAT2* copy number in T_0_ plants indicated that 12 of them have one copy and four have two copies (Table 1). Among the lines, the FA composition exhibited some variations in each non-HFA (Table S1) as well as HFA (Table 1), which can be explained by the heterozygous nature of lesquerella, as it is nearly a wild species, and similar seed-to-seed variations were reported [5]. However, the average levels of each non-HFAs, palmitic (16:0), palmitoleic (16:1), stearic (18:0), oleic (18:1), linoleic (18:2), linolenic (18:3), and eicosenoic (20:1) acids, from transgenic lines are similar to that of wild type (wt) seeds (Table S1). Interestingly, all transgenic lines had increased 18:1OH content ranging from 1.35%–4.08% compared with that of wt at 1.20% (Table 1). Among them, four T_1_ seed populations (lines 1, 3, 4 and 5) showed relative high 18:1OH contents from 3.79%–4.08%. The correlation between 18:1OH content and transgene *RcLPAT2* copy number is weak (*r* = 0.1, *p* = 0.713). About 63% (10/16) of the lines also showed an increase in 18:2OH (Table 1), despite the overall low contents of 18:2OH (0.15%–0.75%) in transgenic lines and wt (0.24%) (Table 1). The correlation between 18:1OH and 18:2OH contents was strong (*r* = 0.91, *p* < 0.00001). With the increase of 18:1OH, we observed a small decrease in 20:1OH levels showing an average of 50.03% compared to 51.50% in wt (Table 1). For 20:2OH, its average level was 3.22%, which is slightly higher than the 3.07% measured for wt (Table 1). Total HFA levels varied from 52.56% (line 8) to 60.44% (line 9) among transgenic lines with an average of 56.08% which is comparable to 56.18% of wt (Table 1). To examine the inheritance of the *RcLPAT2* transgene, we selected hygromycin resistant T_1_ seedlings and examined *RcLPAT2* copy number in those relatively high 18:1OH T_1_ populations. We identified two homozygous lines, 3-1 and 4-5, and produced selfed T_2_ seeds for further analysis. For FA composition, we observed profiles similar to that of T_1_ seeds (Table 2). We note that the 18:2OH content in wt was 0.24% in Table 1 and 0.02% in Table 2. The discrepancy was likely caused by the heterozygous nature of lesquerella seeds and by small sample size used for the analysis (10 seeds per sample for Table 1, and 15 seeds per sample for Table 2). The increased 18:1OH accumulation phenotype in lines 3-1 and 4-5 was inherited into the T_2_ generation, although the levels of 18:1OH in homozygous lines increased only to 5.13% and 4.72%, respectively (Table 2). In general, there was no obvious alteration on growth phenotype in transgenic lines associated with the expression of *RcLPAT2*.

### 2.2. Overexpression of RcLPAT2 Altered the Composition of Hydroxy Fatty Acid (HFA)-Containing Triacylglycerol (TAG) Species and Increased the Percentage of 18:1OH at the sn-2 Position

**Figure 2:**
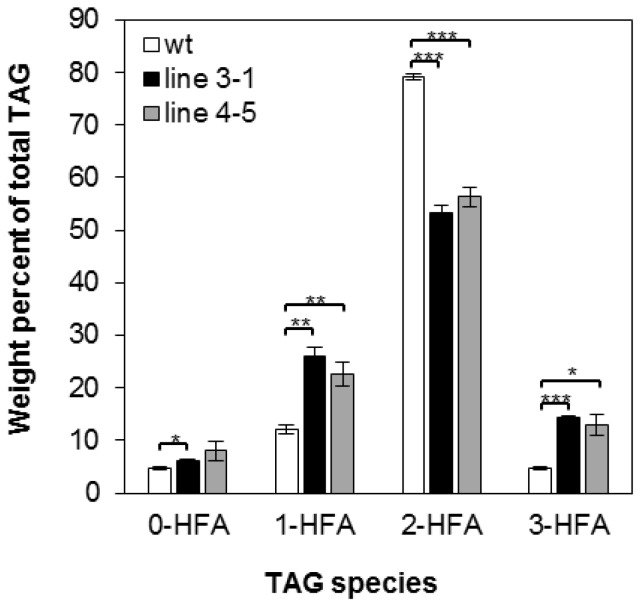
Molecular species composition of TAG. TAG molecular species in seeds of wild type (wt) (open bars) and transgenic line 3-1 (black bars), line 4-5 (grey bars) are measured as a weight percent of total TAG. 0-, 1-, 2-, and 3-HFA represent TAG molecular species with zero, one, two, or three HFAs, respectively (no stereochemistry implied). Triplicates of 50-seed sample were measured for wild type and transgenic lines. The HFAs represent the sum of 18:1OH and 20:1OH. FA numerical symbols are described as in Table 2. The data represent averages of three replicates ± SE. Two-tailed Student’s *t* test. * *p* < 0.05; ** *p* < 0.01; *** *p* < 0.001.

**Figure 3:**
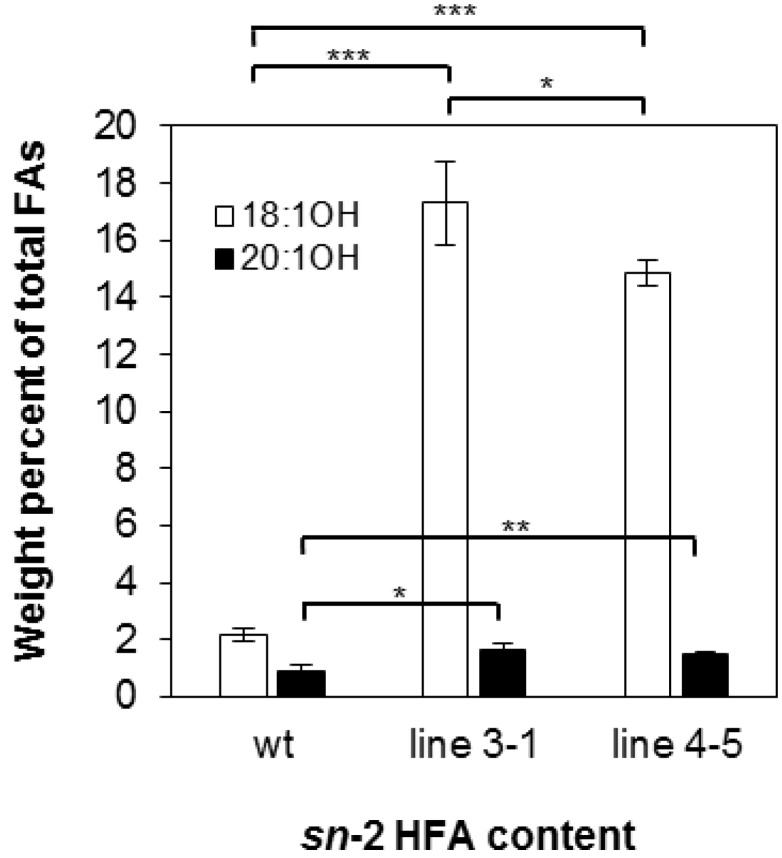
HFA content at the *sn*-2 position of TAG. Ricinoleic acid (18:1OH, open bars) and lesquerolic acid (20:1OH, black bars) at the *sn*-2 position of TAG from wild type (wt) and transgenic line 3-1 and line 4-5 are measured in weight percent of total FA. Triplicates of 50-seed sample were measured for wild type and transgenic lines. The data represent averages of three replicates ± SE. Two-tailed Student’s *t* test. * *p* < 0.05; ** *p* < 0.01; *** *p* < 0.001.

There are four possible molecular species of TAG based on the number of HFA chains esterified to the glycerol backbone: 0-, 1-, 2-, and 3-HFA-TAG. To investigate if there is any change in TAG species composition in the transgenic seeds, we determined the relative amount of each TAG species as a percentage of total TAGs in wt and transgenic lines 3-1 and 4-5. In wt, the relative amount of 0-, 1-, 2-, and 3-HFA-TAG was 4.79%, 12.17%, 79.13% and 4.79% respectively (Figure 2), which are comparable to reported levels [3,56]. In both transgenic lines, there are increases in 0-, 1-, and 3-HFA-TAGs showing 6.13%, 26.06%, and 14.38% in line 3-1 and 8.01%, 22.64%, and 12.93% in line 4-5, respectively (Figure 2). However, we observed that 2-HFA-TAG decreased 29%–33%, showing 53.43% and 56.41% in lines 3-1 and 4-5, respectively (Figure 2). Regiochemical analysis was performed for lines 3-1 and 4-5 to determine whether overexpression of *RcLPAT2* increases the amount of HFAs at the *sn*-2 position. As shown in Figure 3, *sn*-2 18:1OH content increased from 2.19% to 17.29% or 14.85% in lines 3-1 and 4-5 respectively. Only very small increases were observed for *sn*-2 20:1OH from 0.93% to 1.64% or 1.53% in lines 3-1 and 4-5 respectively.

## 3. Discussion

To evaluate the utility of *RcLPAT2* for the engineering of a lesquerella crop producing castor oil, we have produced transgenic lesquerella expressing *RcLPAT2* under the control of a seed specific promoter. Our results indicate that RcLPAT2 enables the incorporation of 18:1OH at *sn*-2 position of LPA which increases the accumulation of 18:1OH and also tri-HFA-TAGs in lesquerella. This is the first successful step in the engineering of lesquerella seeds in order to produce castor oil. However, the overexpression of RcLPAT2 did not lead to an overall increase in total HFAs (Table 1 and Table 2, Figure 2). In fact, we observed small decreases in 20:1OH (Table 1 and Table 2), indicating that in part, the increase in 18:1OH was at the expense of 20:1OH. Since RcLPAT2 and PfKCS3 utilize the same substrate 18:1OH-CoA, it is possible that RcLPAT2 is competing with PfKCS3 for 18:1OH-CoA substrate, limiting the availability of 18:1OH-CoA for the elongation reaction. Therefore, silencing PfKCS3 would block the formation of 20:1OH and allow more 18:1OH-CoA to be directly incorporated into TAGs. In addition, the endogenous PfFAH12 is a bifunctional oleate 12 hydroxylase:desaturase [32], that might not be strong enough to keep up with the demand of 18:1OH-CoA during the seed development. If this is the case, co-expression of a strict 12-hydroxylase gene from castor (*RcFAH12*) [31] or from *Physaria lindheimeri* (*PlFAH12*) [57] might be necessary to boost 18:1OH-CoA availability for incorporation into seed TAGs. On the other hand, lesquerella produces the polyunsaturated FA 18:2 at 8%–9% (Table S1, Table 2), which indicates that it must have an active desaturase *PfFAD2* [58] that coverts 18:1 to 18:2. Since 18:1 is also the substrate for FAH12 to synthesize 18:1OH, suppressing *PfFAD2* activity might increase the availability of 18:1 for FAH12 to produce 18:1OH. Moreover, a microsomal ∆-15 desaturase (PfFAD3) could be silenced to further suppress the conversion of 18:1OH to 18:2OH. Our transgenic lines expressing *RcLPAT2* can be used as genetic backgrounds for studying the effect of additional transgenes such as *RcFAH12* or *PlFAH12* over-expression and/or *PfKCS3/PfFAD2/PfFAD3* silencing in order to develop a castor oil-producing lesquerella.

In transgenic lesquerella expressing *RcLPAT2*, we observed an increase in 0-, 1-, and 3-HFA-TAG levels and a reduction in 2-HFA-TAG species (Figure 2). Since the total HFA content remained unchanged, it is likely that HFAs were redistributed from *sn*-1 and/or *sn*-3 to the *sn*-2 position of TAG due to the limitation of the HFA supply. Regiochemical analysis showed that *sn*-2 18:1OH or 20:1OH increased 6–7-fold or 1.5-fold, respectively (Figure 3). This indicates that *RcLPAT2* allows for a more efficient acylation of 18:1OH than 20:1OH to the *sn*-2 position of TAG *in vivo*.

**Figure 4:**
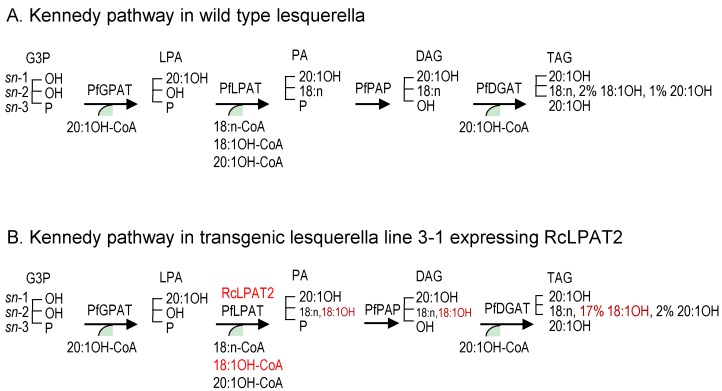
Simplified figure showing the effect of RcLPAT2 on the TAG biosynthetic pathway in lesquerella focusing on the Kennedy pathway. In the Kennedy pathway sequential acylation of a glycerol backbone takes place, by the GPAT, LPAT and DGAT enzymes in order to generate TAG. (**A**) In lesquerella the GPAT and DGAT enzymes have high selectivity for 20:1-OH leading to acylation of the *sn*-1 and *sn*-3 positions of the glycerol backbone with 20:1-OH. LPAT has specificity for non-hydroxylated FAs, preventing the incorporation of significant amounts of HFA (~2%18:1-OH and ~1% 20:1-OH) at the *sn*-2 position of the glycerol backbone (Figure 3); (**B**) In transgenic lesquerella seeds expressing RcLPAT2, e.g., line 3-1, approximately 19% of FAs at the *sn*-2 position of TAG consist of HFAs (~17% 18:1-OH and ~2% 20:1-OH) (Figure 3). However, the total amount of HFAs in the seed oil is not increased, which leads to a decrease in acylation of 20:1-OH the *sn*-1 and *sn*-3 positions (Table 1 and Table 2). This indicates that RcLPAT2 has a high specificity for acylating 18:1-OH to the *sn*-2 position of LPA in lesquerella, which results in an increased amount of tri-HFA-TAGs in transgenic seed oil (Figure 2). 18:n means 18:1, 18:2, or 18:3, described as in Table 2. Other abbreviations are as in Figure 1. Red words show the effect of RcLPAT2.

Although a number of mechanisms may facilitate accumulation of 18:1OH at the *sn*-2 position of TAG, the Kennedy pathway can be perceived as a major route for the channeling of 18:1OH into the *sn*-2 position of LPA by RcLPAT2. Figure 4 summarizes how RcLPAT2 might influence TAG biosynthesis through the Kennedy pathway in transgenic lesquerella seeds. A lesquerella LPAT (PfLPAT) could acylate LPA with HFAs at a low level since we detected about 2% of 18:1OH and 1% of 20:1OH at the *sn*-2 position of wt TAGs (Figure 3 and Figure 4). We have identified a *PfLPAT2* with 92% sequence identity to *AtLPAT2* [59]. Gene expression analysis showed that *PfLPAT2* is ubiquitously expressed in leaf, stem, flower bud, and developing seeds, suggesting it has a house-keeping role in membrane and storage lipid biosynthesis throughout plant life. In lines 3-1 and 4-5, small increase of 2% of 20:1OH at the *sn*-2 position of TAG were detected, however, significant increases of 18:1OH up to 15%–17% were detected (Figure 3 and Figure 4). Thus unlike *PfLPAT2*, *RcLPAT2* has evolved to acylate 18:1OH to the *sn-2* position of LPA in seeds. Our transgenic experiments demonstrate that *RcLPAT2* can be used to produce tri-HFA-TAGs in transgenic plants. In order to further increase the HFA level at the *sn-*2 position, the expression of *PfLPAT2* could be silenced, since substrate competition between endogenous and transgenic acyltransferases can limit the accumulation of UFAs [40,60].

In lesquerella, acyl editing may provide 18:1OH for TAG assembly. Recent studies have suggested that LPCATs are responsible for incorporation of newly synthesized FA into PC (forward reaction), and transferring mFA such as PUFA or HFA produced on PC back to the acyl-CoA pool (reverse reaction) [61,62,63,64] (Figure 1). When the reverse reactions were measured, a ricinoleoyl group at the *sn*-2 position of PC was removed three- to six-fold faster than an oleoyl group by seven LPCATs from five species tested, including lesquerella [64]. Our previous results showed that that there were no HFAs detectable in the PC fraction of total lipids isolated from developing lesquerella seeds [6], it is likely that acyl editing facilitates 18:1OH to be immediately released from PC and then utilized by RcLPAT2 in these transgenic lesquerella lines.

We cannot exclude that the PC-derived DAG pathway may also contributed to the accumulation of 18:1OH at the *sn*-2 position of TAG (Figure 1). There is strong evidence that plants enriched with PUFAs in seed TAG may utilize the PC-derived pathway [62]. Lesquerella seed TAGs contain about 22% PUFAs (18:2 and 18:3) (Table 2), Therefore, it is likely that PC-derived DAGs are utilized in TAG assembly in lesquerella. Overexpression of *RcLPAT2* in developing seeds of lesquerella could enrich the *de novo* DAG pool with *sn*-2 18:1OH-DAGs. The 18:1OH on the *sn*-2 position of the *de novo* DAG could end up at the *sn*-2 position of PC by the action of CPT, generating *sn*-2 18:1OH-PC. If a significant FA flux through PC-derived DAG exists in lesquerella, some of the *sn*-2 18:1OH-PC could be converted by PDCT to *sn*-2 18:1OH-DAG for TAG assembly. The generation of 18:1OH-PC could also provide an opportunity for a ∆-15 desaturase to desaturate 18:1OH-PC to form 18:2OH-PC, resulting in observed small increases of 18:2OH in the transgenic lines.

In conclusion, this is the first demonstration that RcLPAT2 can be used to acylate 18:1OH at the *sn*-2 position of 20:1OH-LPA to generate tri-HFA-TAG, a valuable property for the engineering of a new castor oil-producing crop, such as lesquerella. This work also represents the first successful attempt in genetic engineering of lipid metabolic pathways in lesquerella.

## 4. Materials and Methods

### 4.1. Construction of pGPro4-PnapA-RcLPAT2 Binary Vector

The map and sequence of the pGPro4 vector was described [65]. The promoter sequence of *napA* was cloned into the pGPro4 vector, resulting in *pGPro4-PnapA*. The activity of the *napA* promoter is seed-specific in lesquerella [66]. *RcLPAT2* was PCR amplified from castor cDNA with KOD polymerase (Novagen, Billerica, MA, USA) (Forward: 5′CACCATGGCTGTTGCAGCTGTAGC3′, Reverse: 5′CTAGTCCTGTTTGTTTTCTG3′) and cloned in the pENTR-D-TOPO vector (Life Technologies Ltd., Paisley, UK). Sequencing of *RcLPAT2* showed that there was one nucleotide change from A to G at position 1154 in the cloned *RcLPAT2* in comparison to the *RcLPAT2* sequence (GenBank ID: JQ796917) from the genome sequence in NCBI. This nucleotide change led to an amino acid change (Arg→Gly). This difference is most likely due to the different ecotypes used. To generate *pGPro4-PnapA-RcLPAT2*, *RcLPAT2* in pENTR-D-TOPO was PCR-amplified with gene-specific primers containing Sal I and BstE II restriction enzyme sites (Forward: 5′ACTGAGTCGACGGATCCATGGCTGTTGCAGCTGTAGC3′, Reverse: 5′ACGTGTCTAGAGGTGACCCTAGTCCTGTTTGTTTTCTGC3′) using Roche High fidelity DNA polymerase (Roche, Indianapolis, IN, USA). The PCR product was ligated in the *pGPro4-PnapA* vector digested with Sal I and BstE II.

### 4.2. Plant Transformation, and Estimation of Transgene Copy Number

The lesquerella seeds, WCL-LY2 [67], were kindly provided by Dave Dierig (USDA-ARS, Arid-Land Agricultural Research Center, Maricopa, AZ, USA). Plant transformation was performed using the *Agrobacterium tumefaciens* strain AGL1 [68] carrying the binary vector *pGPro4-PnapA-RcLPAT2*. Tissue culture and plant growth conditions were as described before [10]. We have produced a total of 17 primary transgenic plants (T_0_), plant #6 was lost before maturity. In the greenhouse, we generated selfed T_1_ seeds by hand-pollination between different flowers from the same plant. Transgenic T_1_ plants were obtained by germinating T_1_ seeds in basal medium supplemented with 50 mg/L hygromycin for two weeks and healthy T_1_ seedlings were transplanted into soil for T_2_ seed production. Transgene copy numbers was estimated for T_0_ plants, and T_1_ lines 3-1 and 4-5 using a qPCR method described before [69]. Optimized primer sequences for *RcPLAT2* are 5′TAGGCTGGTCCATGTGGTTTT3′ (forward) and 5′TTCATCCTTGGCCCAGCTT3′ (reverse). For internal control *KCS4/5*, the primer sets are the same as previously reported [69].

### 4.3. Lipid Extraction, TAG Species, Regiochemical and GC Analysis

The GC analysis for Table 1 and Table S1 was performed as described [38]. For Table 2, GC analysis was performed in the following way. One milliliter of hexanes and one mL of methanol containing 2.5% sulfuric acid (*v*/*v*) were added to seeds and incubated for 20 min at 100 °C. The top layer was extracted, dried under nitrogen and dissolved in 100 µL hexanes. GC was performed on a Restek RTX-2330 column (30 m × 0.25 mm inner diameter × 0.2 µm) using the following GC parameters: 120 °C for 1 min, a ramp to 180 °C at 40 °C·min^−1^, a ramp to 190 °C at 7 °C·min^−1^ with a 1 min hold, followed by a ramp to 250 °C at 30 °C·min^−1^ with a 6 min hold. Lipid extractions, analysis of TAG species and regiochemical analysis were performed as described [38]. However, the solvent system used for separating MAGs and 1-HFA-MAGs was based on Bates and Browse, 2011 [20]. In addition, regiochemical analysis was performed on total TAGs instead of separate TAG species as previously described [38]. In brief, TAG species analysis was performed in the following way. 1.5 mg of total lipids from wild type and transgenic lines were run in triplicate on TLC plates (Z292974-1PAK, Sigma-Aldrich Company, Dorset, UK) alongside castor oil and flax oil standards. The TLC plates were first developed for 12 cm in CHCl_3_:methanol:HOAc (93:3:0.5, *v*/*v*/*v*), dried for 15 min, followed by a full development in CHCl_3_:methanol:HOAc (99:0.5:0.5, *v*/*v*/*v*). Lipids were visualized under UV after staining with the fluorescent dye primuline. Bands representing 3-HFA-, 2-HFA-, 1-HFA- and 0-HFA-TAGs were scraped from the TLC plates. The identity of the TAG species in each band was confirmed by measuring the percentage HFAs in each of these TAG fractions by GC (100%, 66%, 33%, and 0% HFAs in the 3-HFA-, 2-HFA-, 1-HFA- and 0-HFA-TAG fractions respectively). The amount of FAs in the 3-HFA-, 2-HFA-, 1-HFA- and 0-HFA-TAG fractions was calculated based on a 15:0 TAG internal standard added to each sample before sample preparation for GC. The relative percentage of each TAG species was calculated in the following way: µg FAs in the individual 3-HFA-, 2-HFA-, 1-HFA- or 0-HFA-TAG fractions/µg FAs in the combined 3-HFA-, 2-HFA-, 1-HFA- and 0-HFA-TAG fractions = relative % of each TAG species as a fraction of total TAG. GC analysis was performed by transmethylating FAs in 1 N methanolic HCl (Sigma-Aldrich Company) for two hours at 85 °C. FAMEs were extracted by adding 500 µL of hexane, and 1 mL KCl, vortexing and centrifugation to separate the hexane layer from the aqueous phase. The hexane layer was collected and dried down under nitrogen and 200 µL BSTFA + TMCS (BSTFA + TMCS 99:1 (Sylon BFT), Sigma-Aldrich Company) was added and incubated at 80 °C for two hours to silylate the FAMEs. The BSTFA + TMCS was evaporated and the silylated FAMEs were dissolved in hexane. Quantification was performed by GC with flame ionization detection on a HP-1MS column (30 m × 0.25 mm i.d. × 0.25 μm, Agilent Technologies LDA UK Limited, Stockport, Cheshire, UK) using the following GC parameters: 50 °C for 1 min, a ramp to 200 °C at 50 °C·min^−1^, followed by a ramp to 325 °C at 3 °C·min^−1^. The identity of each silylated FAME was determined by GC-MS. In contrast to the GC results obtained for Table 1 and Table 2 and Table S1, no 18:2OH and 20:2OH FAs could be detected in the analysis performed for Figure 2 and Figure 3 using GC-MS. Probably the 18:1/18:2-OHs and 20:1/20:2-OHs cannot be resolved using the GC methods we used for these experiments.

## Figures and Tables

**Table 1:** RcLPAT2 copy number in primary transgenic lines and hydroxy fatty acid (HFA) composition of T_1_ seeds.

HFA Content		18:1OH	18:2OH	20:1OH	20:2OH	Total HFA
Wild type		1.20 ± 0.09	0.24 ± 0.06	51.74 ± 0.98	3.07 ± 0.39	56.18 ± 0.48
Transgenics						
ID	Copy number					
Line 1	2	3.79 ± 0.04 ***	0.53 ± 0.04 **	45.64 ± 1.32 *	3.11 ± 0.15	53.08 ± 1.46
Line 2	1	1.74 ± 0.08 *	0.30 ± 0.08	51.43 ± 0.50	3.45 ± 0.21	56.91 ± 0.33
Line 3	1	4.08 ± 0.31 ***	0.74 ± 0.12 *	48.99 ± 0.69	3.60 ± 0.11	57.81 ± 0.34
Line 4	2	3.81 ± 0.06 ***	0.60 ± 0.03 **	50.02 ± 0.48	3.38 ± 0.24	57.81 ± 0.34
Line 5	1	3.97 ± 0.13 ***	0.75 ± 0.20 *	48.35 ± 1.88	3.58 ± 0.31	56.64 ± 1.27
Line 7	1	3.29 ± 0.12 ***	0.34 ± 0.03	51.09 ± 0.22	2.84 ± 0.15	57.56 ± 0.21
Line 8	1	1.42 ± 0.08 **	0.15 ± 0.07	48.24 ± 1.41	2.75 ± 0.20	52.56 ± 1.26
Line 9	1	2.87 ± 0.21 ***	0.53 ± 0.07 *	53.51 ± 0.33	3.53 ± 0.31	60.44 ± 0.36 **
Line 10	1	2.66 ± 0.12	0.43 ± 0.06 *	48.98 ± 1.11	3.09 ± 0.06	55.17 ± 1.00
Line 11	1	1.42 ± 0.08	0.23 ± 0.04	49.57 ± 0.46	3.28 ± 0.09	54.50 ± 0.31 *
Line 12	1	1.35 ± 0.05	0.15 ± 0.03	51.40 ± 1.75	3.26 ± 0.26	56.15 ± 1.58
Line 13	1	2.08 ± 0.11 **	0.29 ± 0.08	47.43 ± 2.07	3.20 ± 0.49	53.00 ± 2.07
Line 14	1	1.97 ± 0.07 **	0.14 ± 0.07	53.09 ± 0.82	2.00 ± 0.13	57.19 ± 0.75
Line 15	1	1.99 ± 0.10 **	0.24 ± 0.13	52.62 ± 0.70	2.93 ± 0.26	57.77 ± 0.47
Line 16	2	1.48 ± 0.04	0.28 ± 0.03	48.96 ± 1.07	3.94 ± 0.10	54.66 ± 1.12
Line 17	2	1.42 ± 0.04	0.23 ± 0.04	51.18 ± 0.59	3.61 ± 0.26	56.44 ± 0.41
Average among lines		2.46 ± 0.26	0.37 ± 0.05	50.03 ± 0.54	3.22 ± 0.11	56.08 ± 0.54

Each HFA composition was measured as percentage of total fatty acids. Triplicates of 10-seed samples were measured for wild type and each independent transgenic line. All data are averages of three measurements ± SE. Fatty acid legend: 18:1OH is ricinoleic; 18:2OH is densipolic; 20:1OH is lesquerolic; and 20:2OH is auricolic acid. Two-tailed student’s *t*-test. * *p* < 0.05; ** *p* < 0.01; *** *p* < 0.001.

**Table 2:** Fatty acid (FA) composition of homozygous T_2_ seeds.

FA	Wild Type	Line 3-1	Line 4-5
16:0	2.75 ± 0.08	3.51 ± 0.28	2.87 ± 0.07
16:1	1.16 ± 0.10	1.02 ± 0.09	1.00 ± 0.17
18:0	3.42 ± 0.83	3.31 ± 0.17	3.43 ± 0.20
18:1	15.19 ± 0.04	14.62 ± 0.30	14.96 ± 0.24
18:2	8.07 ± 0.28	8.43 ± 0.35	8.78 ± 0.48
18:3	13.90 ± 0.05	11.56 ± 0.65	11.40 ± 0.64
20:1	0.88 ± 0.12	0.37 ± 0.03 *	0.68 ± 0.21
18:1OH	1.43 ± 0.07	4.27 ± 0.10 ***	4.23 ± 0.41 **
18:2OH	0.02 ± 0.01	0.86 ± 0.14 *	0.49 ± 0.25
20:1OH	50.35 ± 0.59	47.82 ± 0.89	47.20 ± 0.62 *
20:2OH	2.63 ± 0.18	3.17 ± 0.12	3.50 ± 0.18 *

Each FA was measured as percentage of total FA. Triplicates of 15-seed sample were measured for wild type and transgenic lines. All data are averages of three measurements ± SE. Fatty acid legend: 16:0 is palmitic; 16:1 is palmitoleic; 18:0 is stearic; 18:1 is oleic; 18:2 is linoleic; 18:3 is linolenic; 20:1 is eicosenoic acid; 18:1OH is ricinoleic; 18:2OH densipolic; 20:1OH is lesquerolic; 20:2OH is auricolic acid. Two-tailed student’s *t*-test. * *p* < 0.05; ** *p* < 0.01; *** *p* < 0.001.

## Supplementary Materials

Supplementary materials can be found at http://www.mdpi.com/1422-0067/17/4/507/s1.
