# Supplementary material for: Expression of Castor LPAT2 Enhances Ricinoleic Acid Content at the sn-2 Position of Triacylglycerols in Lesquerella Seed
Source: Int J Mol Sci. 2016 Apr 6;17(4):507. doi: 10.3390/ijms17040507 (PMC4848963; doi:10.3390/ijms17040507)
Supplement: Supplementary file 1 [file ijms-17-00507-s001.pdf]

Supplementary Materials: Expression of Castor LPAT2 Enhances Ricinoleic Acid Content at the *sn*-2 Position of Triacylglycerols in Lesquerella Seed

Grace Q. Chen, Harrie van Erp, Jose Martin-Moreno, Kumiko Johnson, Eva Morales, John Browse, Peter J. Eastmond and Jiann-Tsyh Lin

Table S1. Non-HFA composition of transgenic lesquerella seeds of T_1 lines expressing *RcLPAT2*.

FA	16:0	16:1	18:0	18:1	18:2	18:3	20:1
Wild type	2.04 ± 0.02	1.44 ± 0.01	1.84 ± 0.07	16.85 ± 0.44	8.58 ± 0.29	12.21 ± 0.40	0.87 ± 0.03
Transgenics							
Line 1	3.18 ± 0.29 *	1.94 ± 0.48 **	2.63 ± 0.09 **	16.50 ± 1.03	8.79 ± 0.22	13.06 ± 0.46	0.83 ± 0.05
Line 2	2.08 ± 0.08	1.18 ± 0.06 *	2.00 ± 0.01	16.07 ± 0.64	7.92 ± 0.20	12.83 ± 0.31	1.01 ± 0.01 **
Line 3	1.97 ± 0.08	1.09 ± 0.05 **	2.01 ± 0.02	16.07 ± 0.68	7.84 ± 0.20	12.67 ± 0.48	0.93 ± 0.07
Line 4	1.85 ± 0.02 **	1.19 ± 0.12	2.11 ± 0.09	15.38 ± 0.20 *	7.77 ± 0.19	13.10 ± 0.29	0.98 ± 0.04
Line 5	1.90 ± 0.03 *	1.05 ± 0.08 **	2.12 ± 0.17	16.75 ± 1.42	8.00 ± 0.19	12.56 ± 0.59	0.99 ± 0.03 *
Line 7	2.60 ± 0.06 ***	1.21 ± 0.05 *	1.91 ± 0.11	13.05 ± 0.22 **	9.12 ± 0.40	13.91 ± 0.30 *	0.64 ± 0.03 **
Line 8	2.74 ± 0.15 **	1.40 ± 0.17	2.09 ± 0.06	15.85 ± 0.91	9.26 ± 0.18	15.43 ± 0.05 **	0.66 ± 0.01 **
Line 9	1.76 ± 0.08 *	0.99 ± 0.08 **	1.66 ± 0.04	15.22 ± 0.10 *	6.94 ± 0.23 *	12.10 ± 0.33	0.88 ± 0.02
Line 10	1.97 ± 0.11	1.20 ± 0.23	2.03 ± 0.10	18.57 ± 0.72	7.92 ± 0.13	12.21 ± 0.25	0.93 ± 0.02
Line 11	2.12 ± 0.14	1.40 ± 0.07	2.26 ± 0.11 *	17.82 ± 0.18	8.54 ± 0.27	12.45 ± 0.12	0.92 ± 0.05
Line 12	1.90 ± 0.13	1.40 ± 0.13	2.05 ± 0.09	17.43 ± 1.29	7.63 ± 0.15 *	12.52 ± 0.24	0.91 ± 0.03
Line 13	2.76 ± 0.31	1.57 ± 0.24	2.12 ± 0.14	17.85 ± 1.06	8.87 ± 0.25	12.99 ± 0.33	0.84 ± 0.07
Line 14	2.50 ± 0.02 ***	1.11 ± 0.10 *	1.80 ± 0.14	14.18 ± 0.71 *	10.62 ± 0.10 **	11.93 ± 0.09	0.67 ± 0.04 *
Line 15	2.34 ± 0.11	1.18 ± 0.13	2.06 ± 0.18	14.20 ± 0.16 **	9.35 ± 0.20	12.33 ± 0.49	0.76 ± 0.04
Line 16	2.55 ± 0.37	1.12 ± 0.17	2.35 ± 0.13 *	17.55 ± 0.96	8.39 ± 0.22	12.39 ± 0.69	0.99 ± 0.09
Line 17	2.37 ± 0.05 **	1.19 ± 0.05 **	1.92 ± 0.07	16.74 ± 0.31	7.83 ± 0.22	12.59 ± 0.11	0.92 ± 0.02
Average among lines	2.29 ± 0.10	1.26 ± 0.06	2.06 ± 0.06	16.20 ± 0.38	8.42 ± 0.22	12.82 ± 0.21	0.87 ± 0.03

Each HFA composition was measured as percentage of total fatty acids. Triplicates of 10-seed sample were measured for wild type and each independent transgenic line. All data are averages of three measurements ± SE. Bold numbers represent either the maximum or minimal level of the FAs in each column. Fatty acid legend: 16:0 is palmitic; 16:1 is palmitoleic; 18:0 is stearic; 18:1 is oleic; 18:2 is linoleic; 18:3 is linolenic; 20:1 is eicosenoic acid. Two-tailed student's *t*-test. * $p < 0.05$; ** $p < 0.01$; *** $p < 0.001$.
